# Toward an understanding of the Cdc48/p97 ATPase

**DOI:** 10.12688/f1000research.11683.1

**Published:** 2017-08-03

**Authors:** Nicholas Bodnar, Tom Rapoport

**Affiliations:** 1 Howard Hughes Medical Institute and Department of Cell Biology, Harvard Medical School, Boston, MA, USA

**Keywords:** Cdc48, p97, ATPase

## Abstract

A conserved AAA+ ATPase, called Cdc48 in yeast and p97 or VCP in metazoans, plays an essential role in many cellular processes by segregating polyubiquitinated proteins from complexes or membranes. For example, in endoplasmic reticulum (ER)-associated protein degradation (ERAD), Cdc48/p97 pulls polyubiquitinated, misfolded proteins out of the ER and transfers them to the proteasome. Cdc48/p97 consists of an N-terminal domain and two ATPase domains (D1 and D2). Six Cdc48 monomers form a double-ring structure surrounding a central pore. Cdc48/p97 cooperates with a number of different cofactors, which bind either to the N-terminal domain or to the C-terminal tail. The mechanism of Cdc48/p97 action is poorly understood, despite its critical role in many cellular systems. Recent *in vitro* experiments using yeast Cdc48 and its heterodimeric cofactor Ufd1/Npl4 (UN) have resulted in novel mechanistic insight. After interaction of the substrate-attached polyubiquitin chain with UN, Cdc48 uses ATP hydrolysis in the D2 domain to move the polypeptide through its central pore, thereby unfolding the substrate. ATP hydrolysis in the D1 domain is involved in substrate release from the Cdc48 complex, which requires the cooperation of the ATPase with a deubiquitinase (DUB). Surprisingly, the DUB does not completely remove all ubiquitin molecules; the remaining oligoubiquitin chain is also translocated through the pore. Cdc48 action bears similarities to the translocation mechanisms employed by bacterial AAA ATPases and the eukaryotic 19S subunit of the proteasome, but differs significantly from that of a related type II ATPase, the NEM-sensitive fusion protein (NSF). Many questions about Cdc48/p97 remain unanswered, including how it handles well-folded substrate proteins, how it passes substrates to the proteasome, and how various cofactors modify substrates and regulate its function.

## Structure and function of Cdc48

Proteins of the AAA+ family (ATPases associated with a variety of cellular activities) perform work on a diverse set of macromolecules by using the energy of ATP hydrolysis (for review, see 1, 2). Cdc48/p97 is involved in a number of processes that are united by their requirement for the separation of individual, polyubiquitinated targets from membranes or binding partners, often followed by degradation of these substrates by the 26S proteasome (for review, see 3). Examples of these processes include endoplasmic reticulum (ER)–associated protein degradation (ERAD), an analogous mitochondrion-associated degradation (MAD) pathway, the extraction of stalled nascent chains from the ribosomal exit tunnel, and the removal of various proteins from chromatin. Consistent with a broad role in protein quality control, mutations in human p97 cause several neurodegenerative diseases ^4– 6^.

**Figure 1.**
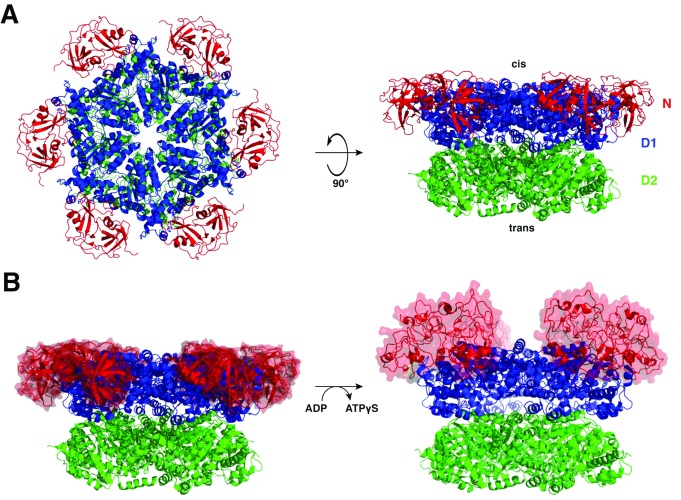
Structure of the Cdc48/p97 ATPase. ( **A**) Cdc48 is a homohexamer, and each monomer comprises an N-terminal (N) domain (red) and two AAA ATPase domains: D1 (blue) and D2 (green). The N-terminal (D1) side of the central pore is referred to as the cis side, and the C-terminal (D2) side as the trans side. ( **B**) ATP binding produces an upward rotation of the N domains into a so-called “up conformation”, in which they are positioned above the plane of the D1 ring. Left, ADP-bound state (PDB code 5FTK); right, ATPγS-bound state (PDB code 5FTN). PDB, Protein Data Bank.

Cdc48/p97 consists of an N-terminal (N) domain and two ATPase domains (D1 and D2). Crystal and cryo-electron microscopy (cryo-EM) structures of p97 show that the D1 and D2 ATPase domains form stacked hexameric rings, with a “cis” and a “trans” side ^7, 8^ ( Figure 1A). Structures determined in the presence of ADP or the ATP analog ATPγS show that the nucleotide state of the D1 ring is coupled to the conformation of the N domains, with the ATP state corresponding to an “up conformation” and the ADP state to a “down conformation” ( Figure 1B). Although these changes were first observed in crystal structures of pathogenic p97 mutants ^9^, recent cryo-EM studies reported similar motions for the wild-type protein ^8, 10^. The two conformations have also been observed in solution by nuclear magnetic resonance ^11^. Individual monomers can adopt the up and down conformations within a given hexamer, allowing a variety of intermediate states ^10^.

Cdc48/p97 cooperates with a variety of protein cofactors (for review, see 12); some of these determine substrate specificity and others trim or extend the ubiquitin chain attached to the substrate. The exact functions of these cofactors are poorly understood. Many cofactors bind to the N domain of Cdc48/p97, but some associate with the C-terminal tail. One of the most important cofactors is the Ufd1/Npl4 (UN) heterodimer, which participates in many Cdc48-dependent processes, including ERAD ^13^. UN is found in all eukaryotes and is essential for their viability. One copy of the heterodimeric cofactor binds to the cis side of a Cdc48 hexamer, with Ufd1 and Npl4 each harboring a binding motif for the N domains ^14– 16^.

Multiple structures of p97 have indicated that the central pore is very narrow or even occluded, leading to the assumption that the polypeptide substrate cannot pass through it. Nevertheless, archaeal Cdc48 homologs and certain mutants of eukaryotic p97 can cooperate with the 20S proteasome to degrade substrate ^17– 19^, which suggests translocation through the pore. This assumption is supported by a recent visualization of a polypeptide chain passing through the central pore of an archaeal Cdc48 complex ^20^. However, the structure lacked the N domain of Cdc48 and the translocating polypeptide was another Cdc48 molecule (that is, a non-physiological substrate). The relevance of the experiments with archaeal Cdc48 for the eukaryotic counterpart is also uncertain because the substrates employed were peptides and non-ubiquitinated proteins, which are poor analogs of Cdc48/p97’s *in vivo* targets. Furthermore, wild-type Cdc48/p97 contains aromatic residues in its conserved D2 pore loop but not in the D1 pore loop, whereas such residues are present in both loops in archaeal Cdc48; the aromatic residues are thought to contact and move the polypeptide substrate through the central pore in other translocating ATPases, such as the eukaryotic 19S proteasome and the bacterial Clp ATPases (for review, see 21). Yet another piece of evidence disfavoring a translocation mechanism for Cdc48/p97 comes from its closest relative, the N-ethylmaleimide–sensitive fusion protein (NSF). Although NSF harbors aromatic pore loop residues in the D1 ring, this ATPase appears to disassemble SNARE (soluble NSF attachment protein receptor) complexes without polypeptide passage through the central pore ^22^, using a single round of ATP hydrolysis to produce relative N domain motions ^23^.

Several specific alternatives to a direct translocation mechanism for Cdc48/p97 have been considered (for review, see 3, 12). In one model, a substrate would transiently enter either the D1 or D2 ring without proceeding through both. Exit of the substrate between the rings, instead of through the end of the pore, has also been suggested. Finally, given that the most obvious structural differences between nucleotide states are found in the N-terminal domains, it has been proposed that Cdc48/p97 might function similarly to NSF, using these N domain conformational changes to exert force on its substrates. A deficiency of all of these models is that they do not explain how the ATPase might generate force to move a polypeptide in a processive manner, which would likely be required to extract substrates from membranes or tightly associated complexes.

Recent results, discussed below, have brought some clarity to the field and indicate that Cdc48 indeed translocates substrates through its central pore rather than using the proposed alternative mechanisms. In the following, we will summarize our knowledge on the function of the Cdc48/UN complex and highlight the many remaining open questions in the field.

## Cdc48 translocates substrates through its central pore

**Figure 2.**
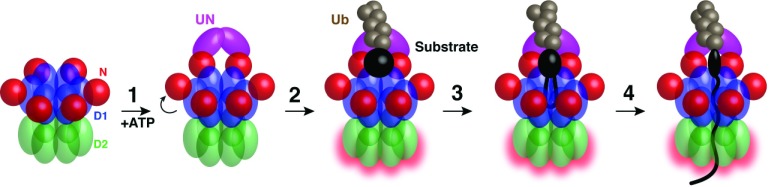
Model for Cdc48 substrate unfolding. The process begins with the N domains in the “down conformation”. In step 1, the N domains transition into the “up conformation” upon D1 ATP binding. Binding of the Ufd1/Npl4 (UN) complex to the N domains renders the complex competent for substrate binding. In step 2, the polyubiquitin chain attached to a substrate binds to the UN complex. The D1 domain stays in the ATP-bound state with the N domains in the “up conformation”. At the same time, the ATPase rate of the D2 domains is increased (red outline). In step 3, the substrate inserts into the central pore. In step 4, ATP hydrolysis by the D2 domains unfolds the substrate by pulling it through the pore to the trans side.

The recent progress on the function of the Cdc48/UN complex was achieved by the use of purified components and model substrates carrying ubiquitin chains with Lys48 linkages ^24, 25^. These and previous results led to a model for the individual steps of Cdc48 function ( Figure 2). In the first step, the UN complex binds to the cis side of Cdc48 ( Figure 2, step 1). Next, the UN complex binds the polyubiquitin chain (step 2). In agreement with previous conclusions ^26^, the polyubiquitin chain has to be of sufficient length (a minimum of five ubiquitin moieties) and is recognized by the UN complex rather than Cdc48 itself. Polyubiquitin binding stimulates the overall ATPase activity of the Cdc48 complex. Experiments with mutations that specifically eliminate ATP hydrolysis in D1 or D2 showed that substrate actually decreases the ATP hydrolysis rate of the D1 domain and increases that of the D2 domain. ATP hydrolysis in D2 moves the substrate polypeptide entirely through the central pore (step 3). This was demonstrated by photocrosslinking to internal amino acid positions in the D1 and D2 rings of Cdc48 and by the use of a fusion of Cdc48 with the protease domain of FtsH, which led to degradation of the substrate. Cdc48 generates a pulling force, as shown by the unfolding of fluorescent proteins in two different studies ^24, 25^. The substrate might insert as a loop into the central pore (step 4), although insertion of the substrate’s C or N terminus has not been excluded. Which portion of the polypeptide initially inserts into the pore may be substrate-specific and dependent on the location of the ubiquitin attachment site, as proposed for the proteasome ^27^.

Interestingly, two recent studies found that hydrolysis by the D1 domain was unnecessary for substrate unfolding and pore translocation ^24, 25^. In the case of yeast Cdc48, a moderate decrease in unfolding capacity was observed when hydrolysis in D1 was eliminated, whereas with human p97 no change at all was observed. Given that ATP hydrolysis in D1 is inhibited by substrate binding, the D1 domain probably stays in the ATP-bound state throughout translocation of a polypeptide chain, keeping the N domain in the “up conformation” (steps 1–4). In contrast, the D2 domain hydrolyzes ATP many times to move the polypeptide through the central pore. Consistent with the D2 domain doing all the work, only the D2 ring contains the canonical aromatic pore ring residues. The schemes in Figure 2 are likely a simplification because the ATPase subunits in a ring may be in different nucleotide states. Thus, in reality, probably only some of the six subunits of the D1 or D2 rings execute the described nucleotide binding and hydrolysis steps simultaneously.

## Substrate release from the Cdc48 complex

**Figure 3.**
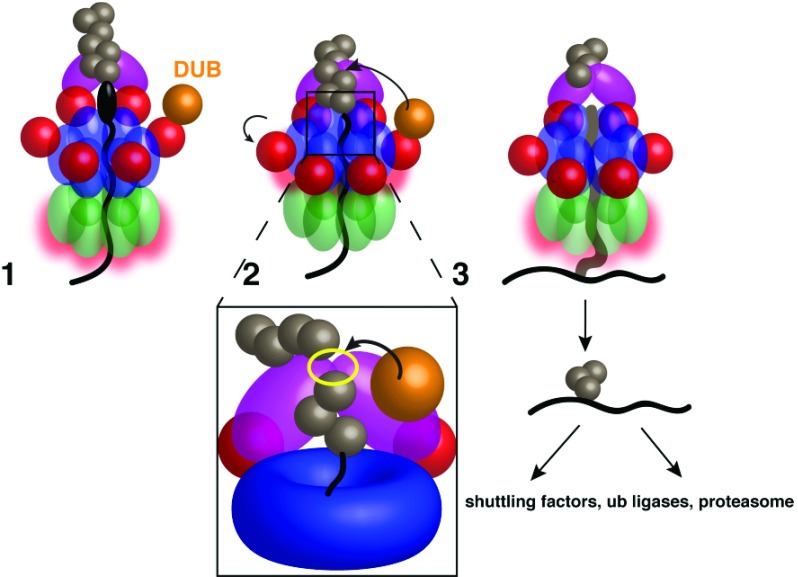
Model for substrate release from Cdc48. Stage 1: The substrate is pulled through the double-ring ATPase, but the polyubiquitin chain is bound to the Ufd1/Npl4 (UN) complex at the cis side, preventing full substrate translocation through the pore, even with continued D2 ATP hydrolysis. A deubiquitinase (DUB) bound to the N domain cannot access the polyubiquitin chain while D1 is in the ATP-bound state and the N domains are in the “up conformation”. Stage 2: D1 hydrolyzes ATP after the substrate has initiated or completed translocation, moving the N domains into the “down conformation” and allowing DUB access to the ubiquitin chain. Inset: The likely Ub chain cleavage site is between the UN binding site (yellow outline) and the central pore; any Ub moieties proximal to the cleavage site will be translocated. Stage 3: After polyubiquitin cleavage, the oligoubiquitinated substrate proceeds fully through the pore. The ubiquitin moieties probably refold after translocation, and the substrate is transferred to downstream factors.

Full translocation of a substrate peptide through the Cdc48 ATPase rings would be counteracted by the association of the attached polyubiquitin chain with the UN cofactor at the cis side ( Figure 3, stage 1). Indeed, experiments with purified components confirmed that substrate release from the Cdc48 complex does not occur spontaneously but rather requires a deubiquitinating enzyme (DUB) ^24^. Addition of Otu1, a DUB that binds to the N domain of Cdc48 via its Ubx-like domain ^28^, resulted in trimming of the polyubiquitin chain and substrate release (stages 2 and 3). Surprisingly, only a minority of the released substrate molecules had lost all ubiquitins; most retained an oligoubiquitin chain with up to 10 ubiquitin molecules. Photocrosslinking experiments showed that ubiquitin was moved through the central pore. Furthermore, a Cdc48 mutant with a reduced unfolding rate retained oligoubiquitinated substrates, indicating that these are slowly translocated through the ATPase. Taken together, these results suggest that the oligoubiquitin chain is also pulled through the central pore ( Figure 3, stage 3). The translocated moieties would be those located between the Otu1 cleavage site and the substrate attachment site ( Figure 3, inset). These results imply that the translocating ubiquitin molecules are sequentially unfolded and that at least two polypeptide strands can be accommodated inside the pore, as demonstrated for other AAA ATPases ^29– 31^. Thus, the pore must be wider during translocation than seen in current structures of the resting ATPase. Another question is whether branched ubiquitin chains, which enhance unfolding efficiency ^25^, are translocated in a similar fashion as single chains.

Although ATP hydrolysis by the D1 domain is not required for substrate unfolding, it is critical for the deubiquitination step ( Figure 3); a D1 mutant that cannot hydrolyze ATP prevented substrate release ^24^. Because the ATP-bound state of the D1 domain corresponds to the “up conformation” of the N domains, one possible interpretation is that the N domains restrict DUB access to the ubiquitin chain, permitting deubiquitination only after D1 ATP hydrolysis. However, this hypothesis will require further validation. In addition, experiments with D2 mutations showed that both ATP binding and hydrolysis by the D2 domain are required for efficient substrate release, suggesting that the deubiquitination step is delayed until translocation has been initiated.

Otu1 is a conserved protein (called YOD1 in mammals), and expression of an enzymatically inactive mutant blocks Cdc48/p97 function *in vivo* and leads to the accumulation of polyubiquitinated proteins on the Cdc48/p97 complex ^26, 32^. However, deletion of Otu1 in *Saccharomyces cerevisiae* has no effect on ERAD ^26^, indicating that other DUBs can replace it. Whether deubiquitination by Otu1 or other DUBs is required for all substrate release events remains to be determined.

## Transfer of substrates from Cdc48/p97 to the proteasome

Cdc48/p97 is generally thought to function upstream of the proteasome, handling substrates that ultimately are destined for proteasomal degradation. The recent *in vitro* experiments show that many of the substrate molecules released from Cdc48 contain four or more ubiquitin molecules ^24^, which would be a suitable targeting signal for the proteasome ^33^, provided that ubiquitin molecules emerging from the D2 ring rapidly refold. At least in the case of ERAD, substrates do not directly bind to the proteasome but rather first interact with Rad23 or Dsk2 ^34, 35^. These proteins seem to serve as “shuttling factors” by harboring both ubiquitin- and proteasome-binding domains. However, the *in vitro* experiments show that some substrates released from Cdc48 bear ubiquitin chains that are too short to directly bind to the proteasome or shuttling factors, and in these cases the Cdc48-associated “E4” ubiquitin ligase Ufd2 might need to extend the oligoubiquitin chains before handoff to the downstream components ^36^. Released substrates that contain exposed hydrophobic segments may require the additional activity of a “holdase” complex anchored by Bag6, which reduces aggregation of substrates destined for degradation ^37^. Finally, some substrates may be transferred directly from Cdc48 into the 20S proteasome without involvement of the 19S subunit ^17– 19^, although evidence for such an interaction in eukaryotes is lacking ^38^. Interestingly, in the absence of Ufd3, a cofactor that binds to the C-terminal tail of Cdc48, ubiquitin is degraded abnormally quickly by the proteasome in yeast cells ^39, 40^. One possibility is that Ufd3 antagonizes the Cdc48-20S interaction, preventing the degradation of unfolded ubiquitin molecules emerging from the D2 ring of Cdc48. Alternatively, Ufd3 may somehow facilitate the refolding of ubiquitin molecules that emerge from the D2 ring.

## A model for Cdc48 function in ERAD

**Figure 4.**
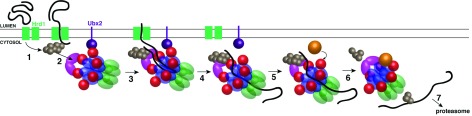
Model for Cdc48 function in endoplasmic reticulum–associated protein degradation. In step 1, a segment of a misfolded luminal substrate is moved into the cytosol and polyubiquitinated by Hrd1. In step 2, the Cdc48 complex is bound to the cytosolic face of the endoplasmic reticulum via an interaction of its N domain with Ubx2. The complex recognizes the polyubiquitin chain via Ufd1/Npl4 (UN). In step 3, Cdc48 uses ATP hydrolysis in the D2 domains to extract the substrate from the membrane, translocating the polypeptide through its central pore. In step 4, Cdc48 completes translocation and eventually diffuses away from Ubx2, together with the bound substrate. In step 5, a deubiquitinase (DUB) such as Otu1 binds to the newly vacant N domain. In step 6, the D1 domains hydrolyze ATP, moving the N domains to the “down conformation” and allowing trimming of the ubiquitin chain. The oligoubiquitinated substrate then is released from the trans side of the pore. In step 7, the substrate is transferred to downstream factors, eventually arriving at the proteasome.

In ERAD, the Cdc48 complex extracts polyubiquitinated, misfolded proteins from the ER membrane. This process can be reproduced with proteoliposomes and purified proteins ^26^, demonstrating that no other component is required. Based on the recent insights into the mechanism of Cdc48 ( Figure 2 and Figure 3), a refined model can be proposed for the function of the ATPase in ERAD ( Figure 4). After retrotranslocation of a substrate segment across the ER membrane, a Lys48-linked polyubiquitin chain is appended to the substrate by an ER-resident ubiquitin ligase (for example, Hrd1) (step 1). Next, the polyubiquitin chain binds to the Cdc48 complex (step 2), which is recruited to the ER membrane via an interaction of Cdc48’s N domain with the membrane protein Ubx2 ^41^. Cdc48 processively extracts the substrate from the ER membrane, passing the entire polypeptide through its central pore (step 3). Once the substrate is fully removed from the ER, Cdc48 dissociates from the Ubx2 anchor, diffusing away from the membrane with its associated substrate (step 4). This dissociation step may be triggered or simply stochastic but would be required to free an N domain binding site for a DUB, such as Otu1 (step 5), as these proteins bind to the same site ^26^. Hydrolysis of ATP by the D1 domain exposes the ubiquitin chain to the DUB, resulting in chain trimming and translocation of the remainder of the oligoubiquitinated substrate through the Cdc48/p97 pore (step 6). Finally, the unfolded substrate is retrieved by one of the accessory factors discussed above and passed on to the proteasome (step 7). In the case of glycosylated ERAD substrates, the N glycans probably are moved through the central pore and removed after translocation, as the cytosolic N-glycanase Png1 binds to the C-terminus of Cdc48 ^42, 43^.

## Open questions and future directions

Many questions about Cdc48 function remain unresolved, including the mechanism of initiation of substrate translocation, the cooperation between ATPase domains, substrate transfer to the proteasome, the functional consequence of disease mutations, and the roles of various cofactors.

One important issue is how Cdc48 can initiate translocation on tightly folded proteins. Some Cdc48 substrates, such as those in ERAD, may be misfolded or loosely folded to begin with. However, Cdc48, unlike the 19S proteasome ^44^, seems not to require a flexible initiation region ^45^. It is unclear how a well-folded substrate bound to the cis side of the ATPase could reach into the pore and contact the D2 pore loops, which are responsible for powering translocation. Likely, the answer will come from cryo-EM structures of the Cdc48/UN complex with polyubiquitinated substrate. These structures could clarify whether substrate binding leads to partial unfolding and whether the central pore widens during translocation to accommodate multiple strands. Most importantly, they would show whether Cdc48 adopts a staircase arrangement of its ATPase domains, as proposed for archaeal Cdc48, the 19S proteasome, and NSF ^20, 22, 46, 47^, or whether it remains in the double-ring conformation seen in the inactive states. Finally, such structures might also shed light on whether the ubiquitin chain indeed is sterically shielded from DUB enzymes when the N domains are in the “up conformation”.

Another unresolved question concerns the communication between the ATPase domains. The mechanism by which substrate binding at the cis side of the double ring is converted into ATPase stimulation at the D2 (trans) side is unclear, although the D1-D2 linker appears to be important for inter-ring communication ^48^. In addition, communication seems to occur in the reverse direction because the activity of Otu1, bound to the cis side, is influenced by the nucleotide state of D2 ^24^. Furthermore, mechanisms of intra-ring ATP hydrolysis coordination in the context of cofactor and substrate binding remain to be investigated. For example, do individual subunits in a ring hydrolyze ATP in a sequential or stochastic manner?

A third area of future work concerns the different mechanisms of proteasomal transfer discussed above. Perhaps one of these pathways (ubiquitin refolding followed by binding to the 26S particle, direct transfer into the 20S particle, extension of ubiquitin chains, or chaperoning by shuttling factors) is preferred *in vivo* and others are used only as necessary. Alternatively, certain substrate types such as membrane proteins might require particular transfer pathways. A third possibility is that individual Cdc48 pathways (for example, ERAD, MAD, and chromatin-based functions) involve specific accessory cofactors that bias substrates toward one of the transfer pathways. Reconstitution of Cdc48-dependent proteasomal degradation *in vitro* would be the first step toward solving these issues. Of note, some substrates, such as the Spt23 ^49^ and Nrf1 ^50^ transcription factors, presumably refold after translocation through Cdc48 to exert their function. How such substrates are spared from proteasomal degradation requires further investigation.

The reasons why p97 disease mutations are deleterious remain ambiguous. Most of these mutations localize to the interface between the D1 and N domains ^51^ and might perturb coordination between ATP hydrolysis and N domain movements ^52, 53^, which could alter the dynamics of substrate deubiquitination and release by Otu1 and other DUBs. Disease mutants also exhibit altered interactions with certain cofactors ^54, 55^. Interestingly, p97 disease mutants are more efficient than the wild-type protein in unfolding substrates ^25, 56^. How hyperactive unfolding, altered cofactor interactions, improper regulation of substrate release, or some combination of these contribute to disease development will be important future topics.

The precise function of most cofactors also remains to be clarified. Although some cofactors seem to function solely to recruit Cdc48/p97 to particular cellular locations, others are more enigmatic. For example, why does deletion of Ufd3 result in depletion of cellular ubiquitin pools? How do shuttling factors such as Rad23 and Dsk2 avoid interaction with polyubiquitinated substrates before they are processed by Cdc48/UN? When and where does the ubiquitin ligase Ufd2 extend polyubiquitin chains, and why does this protein seem to bind to the C-terminal tail in yeast when its mammalian homolog binds to the N domain? ^57^ Which DUBs, in addition to Otu1, can release substrates from Cdc48, and are they preferred in specific contexts?

The demonstration that Cdc48/UN is an unfoldase that acts via a translocation mechanism clarifies a long-standing question in the field but now demands further investigation into why specific substrates require Cdc48 for unfolding, how that unfolding is triggered and regulated, and how Cdc48 is integrated via its cofactors into a wide array of pathways.
